# The Use of Random Projections for the Analysis of Mass Spectrometry Imaging Data

**DOI:** 10.1007/s13361-014-1024-7

**Published:** 2014-12-19

**Authors:** Andrew D. Palmer, Josephine Bunch, Iain B. Styles

**Affiliations:** 1 PSIBS Doctoral Training Centre, University of Birmingham, Edgbaston B15 2TT Birmingham, UK; 2 Zentrum für Technomathematik, Fachbereich 3, Universität Bremen, Postfach 33 04 40, 28334 Bremen, Deutschland; 3 National Physical Laboratory, Hampton Road, Teddington, TW11 0LW Middlesex, UK; 4 School of Pharmacy, University of Nottingham, University Park NG7 2RD Nottingham, UK; 5 School of Computer Science, University of Birmingham, Edgbaston B15 2TT Birmingham, UK

**Keywords:** Random projection, Mass spectrometry imaging, Informatics, Segmentation, Digital histology, Dimensionality reduction, Data processing

## Abstract

The ‘curse of dimensionality’ imposes fundamental limits on the analysis of the large, information rich datasets that are produced by mass spectrometry imaging. Additionally, such datasets are often too large to be analyzed as a whole and so dimensionality reduction is required before further analysis can be performed. We investigate the use of simple random projections for the dimensionality reduction of mass spectrometry imaging data and examine how they enable efficient and fast segmentation using *k*-means clustering. The method is computationally efficient and can be implemented such that only one spectrum is needed in memory at any time. We use this technique to reveal histologically significant regions within MALDI images of diseased human liver. Segmentation results achieved following a reduction in the dimensionality of the data by more than 99% (without peak picking) showed that histologic changes due to disease can be automatically visualized from molecular images.

**Electronic supplementary material:**

The online version of this article (doi:10.1007/s13361-014-1024-7) contains supplementary material, which is available to authorized users.

**Graphical Abstract:**
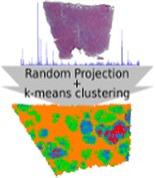
ᅟ

## Introduction

The determination of molecular profiles from individual tissue types is central to the understanding of their biological function, and direct chemical analysis of tissue using mass spectrometry imaging (MSI) is an established tool for determining profiles encompassing a broad range of molecules within a single imaging experiment [7, 29]. One route to producing molecular profiles is to group similar tissue regions according to the similarity of their mass spectra, and to extract an average spectrum for each group. Manually identifying distinct tissue types is difficult and requires a histologic expert [2, 8], so several groups have examined automated segmentation methods for group identification to provide an unsupervised and reproducible scheme for the analysis of data [9, 7, 29, 24].

These clustering methods were shown to be useful in MSI for extracting distinct histologic regions [2], separating tumor from normal tissue [9], and for three-dimensional visualization of tissue structures [29]. Other sophisticated approaches have been developed for viewing data heterogeneity [14] and provide powerful tools for the visualization of trends within mass spectrometry images. A specific advantage of segmentation is that all tissue regions within clusters have similar spectra by construction, and so molecular profiles for the corresponding tissue types can be computed. These profiles can be used to identify discriminatory, characteristic, or spatially co-varying molecules. This work addresses two issues that restrict the application of automated processing of mass spectra; first, the number of peaks that can be processed, and second, the ability to perform data-processing in real time whilst data is still being collected.

Automated segmentation identifies clusters of similar spectra using a ‘distance’ metric to quantify spectral similarity. A significant issue when calculating distance metrics for mass spectra is the *dimensionality* of the data which, in the case of mass spectra, is equal to the number of *m/z* values being considered. In a time-of-flight spectrum, this could be more than 100,000 mass bins, and could be millions for high-resolution instruments. High dimensionality negatively affects accuracy of distance metrics as the *relative* differences between distances tends to zero (so all spectra are measured as being equally different to each other). Two factors compound this problem even further in MSI: the number of samples (pixels) is nearly always much lower than the dimensionality, and the covariance of samples introduces redundancy into the data and effectively reduces the sampling rate further. Dimensionality reduction methods are frequently used to allow accurate distance calculations [28] by removing this redundancy between spectral channels. This allows the accuracy and speed of cluster formation to be improved [10], either by choosing a small number of ‘important’ measurements or by a transformation of the data. A common approach involves a linear transformation of the data by projection onto a low dimensional basis which, if constructed correctly, will preserve key relationships between samples and allow analyses such as segmentation to be performed on the projected data [19, 24]. Unfortunately dimensionality reduction often carries a high computational cost or requires multiple passes through the data in order to extract a meaningful set of measurements. Commonly used methods such as principal component analysis and non-negative matrix factorization have been shown to be effective on mass spectrometry images [16] but have the distinct disadvantage of requiring the basis to be calculated from the data. This usually means the whole dataset needs to be collected and loaded into memory to compute the basis, which prevents real-time analysis and may be impossible for very large datasets, in which case a preliminary stage of data reduction is required [21, 26]. The issue of coping with the size of mass spectrometry imaging data has been noted for almost as long as the field has existed [9, 1]. Most workflows described in the literature go through a multi-stage process of peak identification and feature selection that can require extensive processing and completely removes some peaks from the subsequent analysis [21, 1, 14].

The quality of segmentation is then dependent on the quality of the peak picking, which can require extensive tuning for specific mass spectrometers, sample preparation techniques, and datasets [11].

An alternative approach uses a pseudo-basis composed of randomly drawn vectors onto which the data is projected [30, 6]. The central idea is that projections onto a collection of such random vectors can be shown to extract almost mutually independent information and so a set of these vectors will capture the essential features of the data [6]. The random basis itself is formed independently of the data and so removes a major computational hurdle. Random projections have been shown to preserve patterns within the data, including distances and angles between data points [19], making them useful for dimensionality reduction in areas including image processing and text mining [6]. Previously, applications in the processing of mass spectrometry data were to compare individual spectra against a database [31] and to form orthonormal approximate bases for mass spectrometry imaging compression [24]. The importance of using memory-efficient data processing is well-known [26] and the random projection algorithm can be implemented in a memory-efficient manner to avoid loading the whole dataset at once.

In this paper, we investigate the use of random projections to enable efficient image segmentation for the identification of spatial features in mass spectrometry images without requiring peak picking or other data reduction stages.

## Experimental

### MALDI MSI of Human Liver

The mass spectrometry dataset used in this work consists of a MALDI mass spectrometry image acquired from a section of diseased human liver suffering from non-alcoholic steatohepatitis (NASH). This dataset has previously been used to demonstrate novel mass spectrometry image visualization methods [14], and a full description of the imaging methodology can be found in the supporting information of that paper. A brief summary is presented here.

#### Tissue Handling

Samples were collected from patients undergoing liver transplantation or tumor resection surgery at The Queen Elizabeth Hospital in Birmingham, with local research ethics committee approval (NHS Walsall LREC) and written informed patient consent during transplantation surgery. All samples were rapidly processed and snap-frozen in liquid nitrogen prior to storage at –80°C.

#### Sectioning

Serial tissue sections were obtained at 5 μm using a cryostat (model OFTF; Bright Instruments, Cambridge, UK) either onto steel MALDI target plates (ABSciex, Warrington, UK) for mass spectrometry or glass slides destined for H&E staining.

#### H&E Staining

Tissue architecture was visualized by routine hematoxylin and eosin (H&E) staining and optical microscopy.

#### MALDI Imaging

Fifteen mg mL^–1^ ***α***-cyano-4-hydroxycinnamic acid (CHCA) in 80% CH_3_OH, 0.1% trifluoroacetic acid (TFA) was applied to the sample and MALDI plate using an artist airbrush (Draper, Hampshire, UK) with Badger Airbrush propellant (Badger, IL, USA), approximately 10 mL of matrix solution was dispensed in total. MALDI TOF MS analysis was carried out on a hybrid quadrupole time of flight mass spectrometer (QStar XL, Analyst QS 1.1, and oMALDI 5.1, ABSciex, Warrington UK) equipped with a Nd:YVO_4_ (355 nm, 5 kHZ, Elforlight: SPOT-10-100-355; Elforlight, Daventry, UK) fiber delivered (100 μm core diameter) diode pumped solid state laser, providing a mass resolving power of >6000 at *m/z* 643. Spectra were acquired in positive ion mode in the mass range *m/z* 600–950 with a spatial resolution of 100 μm in both x and y directions.

#### Data Processing

Mass spectrometry images were extracted from the proprietary instrument format (.wiff) to the imzML format [converting to mzML using AB SCIEX MS Converter (ver. beta 1.1; ABSciex, Warrington UK), then to imzML using imzMLConverter (ver. 1.0, www.imzMLConverter.co.uk [25])]. The imzML parser included with imzMLconverter was used to load individual spectra into MATLAB (Mathworks, Nantucket, MA, USA).

### Random Projection

A mass spectrometry image is represented as a 2D data matrix ***X***_*m* × *n*_ where *m* is the number of spectral channels and *n* is the number of pixels, typically *m* ≫ *n*. The random projections are implemented by constructing a matrix ***Q***_*k* × *m*_, where *k* is an integer controlling the number of projections. Each element of ***Q*** is drawn from a zero-mean normal distribution with unit standard deviation (*N*(0, 1)) [15] and each row of ***Q*** corresponds to a random direction in spectral space onto which the data is projected by calculating ***A*** = ***QX***, giving a projection score matrix ***A***_*k* × *n*_. By setting *k* < *m* the dimensionality is reduced following projection.

We note that this can be implemented in a memory-efficient manner as the spectra are projected independently so that the full data matrix ***X*** does not need to be loaded into memory in its entirety.

### Segmentation

#### k-Means Clustering

Segmentation was performed using the *k*-means algorithm implemented as the function kmeans in the MATLAB Statistics Toolbox (MATLAB R2009a). The algorithm is initialized by specifying a number of clusters, then arbitrarily allocating each data point to one of the clusters. The algorithm then proceeds iteratively by calculating the geometric center of each cluster and then allocating each data point to the cluster whose centroid is closest according to the Euclidean distance in the spectral space. The algorithm ends when membership of the clusters stabilizes. For visualization, every member of each cluster is assigned the same color (allowing spatially disconnected regions to have the same color), and a segmentation map is formed showing the class of each pixel.

### Code Implementation

The random projection algorithm was implemented in MATLAB and demonstration code is provided in the Supporting Information.

## Results

We have evaluated the use of random projections for dimensionality reduction in MSI on a benchmark dataset whose histologic features have previously been identified using several approaches to MSI visualization [14]. A second demonstration on a publicly available mouse brain dataset that was included in the supplementary information of Race et al. (2013) [26] is contained in the Supporting Information (see Supplementary Figure S2).

### Mass Spectrometry Imaging of Human Liver

The benchmark dataset consists of a MALDI mass spectrometry image acquired from a section of diseased human liver suffering from non-alcoholic steatohepatitis (NASH). The dataset contains 12,325 pixels each with an associated spectrum in 33,725 *m/z* channels, resulting in a raw data size ≈3 GB.

NASH disease is characterized by the accumulation of fat within liver hepatocytes (steatosis) and in a proportion of patients this is followed by the development of necro-inflammatory activity that leads to cirrhosis [17, 13]. The development of liver cell ballooning and inflammation (steatohepatitis) determines whether a patient progresses to irreversible liver damage and fibrosis [18] and can currently only be identified by histologic examination [4].

**Figure 1:**
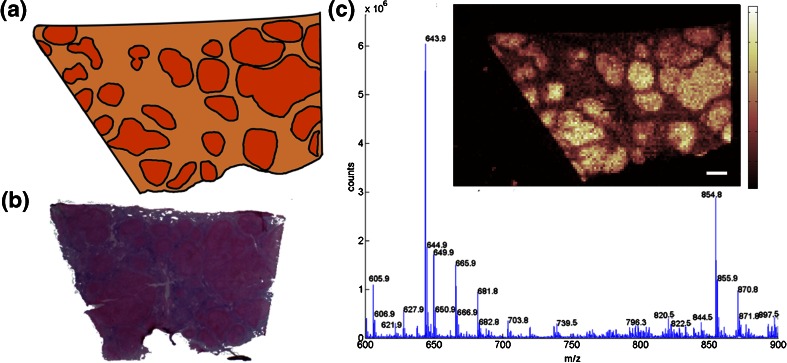
(**a**) Schematic of the liver image showing characteristic histology of NASH disease including fibrotic tissue (pale) and enlarged hepatocytes (dark), (**b**) H&E macroscopy image that also shows the presence of enlarged hepatocytes. (**c**) Mean mass spectrum from the tissue showing multiple peaks within the lipid region. The example ion image (*m/z* 796.5 ± 0.25) shows greater intensity in parenchymal areas of hepatocytes separated by bands of fibrotic tissue with much lower signals (scale bar 1 mm)

The major histologic features that are commonly seen within NASH diseased tissue are visible in this dataset (Figure 1). The normal appearance of the liver has been severely deformed by the ballooning hepatocytes which are separated by regions of fibrotic connective tissue. Hepatocytes would not normally be individually visible on this scale but enlargement attributable to NASH makes them clearly identifiable. Histologic examination and other visualization approaches [14] suggest that some of the hepatocytes in large clusters in the upper right of the image may be regenerating. A comparative image taken from a section of normal liver is shown in Supplementary Figure S1 and has the expected smooth appearance on this scale.

Spectra were averaged from the tissue and a substantial number of peaks were visible within the *m/z* range 700–900, which is known to correspond to the masses of multiple lipids (Figure 1). Manual inspection of the data revealed several peaks that produced ion images that reflected the tissue histology; an arbitrary example from a peak of low intensity in the mean spectrum is shown in Figure 1. To obtain a rough estimate of the spectral complexity of the dataset, peak picking was applied to the mean spectrum (maximum-window peak detection [20]), which returned >900 peak centroids, the majority of which do not correspond to *m/z* values associated with CHCA matrix [27]. This gives an indication of the degree to which the data can potentially be reduced but applying peak detection to all spectra within an image, and aligning the results is computationally intensive [1]. As the random projections are data-independent, they can be generated without the dataset in memory and applied piece-wise to one pixel at a time.

### Random Projection of MSI

The random projection of the data onto the *k* random vectors that make up ***Q*** creates *k* vectors, each of which randomly samples over the whole *m/z* range. Each projection therefore captures a randomly weighted linear combination of all *m/z* channels and, thus, samples the full range of chemical information present. As the sampling is random, there is no a priori way of knowing what chemical information will be captured by a particular projection, and direct analysis of single projections is unlikely to be informative, but by taking many projections, all of the information can be captured with very high probability.

It is also important to note that the projection vectors are chosen from a zero-mean Gaussian so they contain values of both signs. Accordingly, the scores also have both positive and negative values, which present some difficulties in relating the projection intensities to their physical origin.

In this work, projections are applied to the data sequentially, loading each column of ***X*** in turn and forming the *k* projections for each pixel in turn. The time it takes to project a spectrum (150 random projections of a single spectrum takes ≈0.1 s) is lower than the data acquisition time (≈0.5 s), which makes this potentially usable for real-time analysis of data during the acquisition process.

### Segmentation from Random Projections

**Figure 2:**
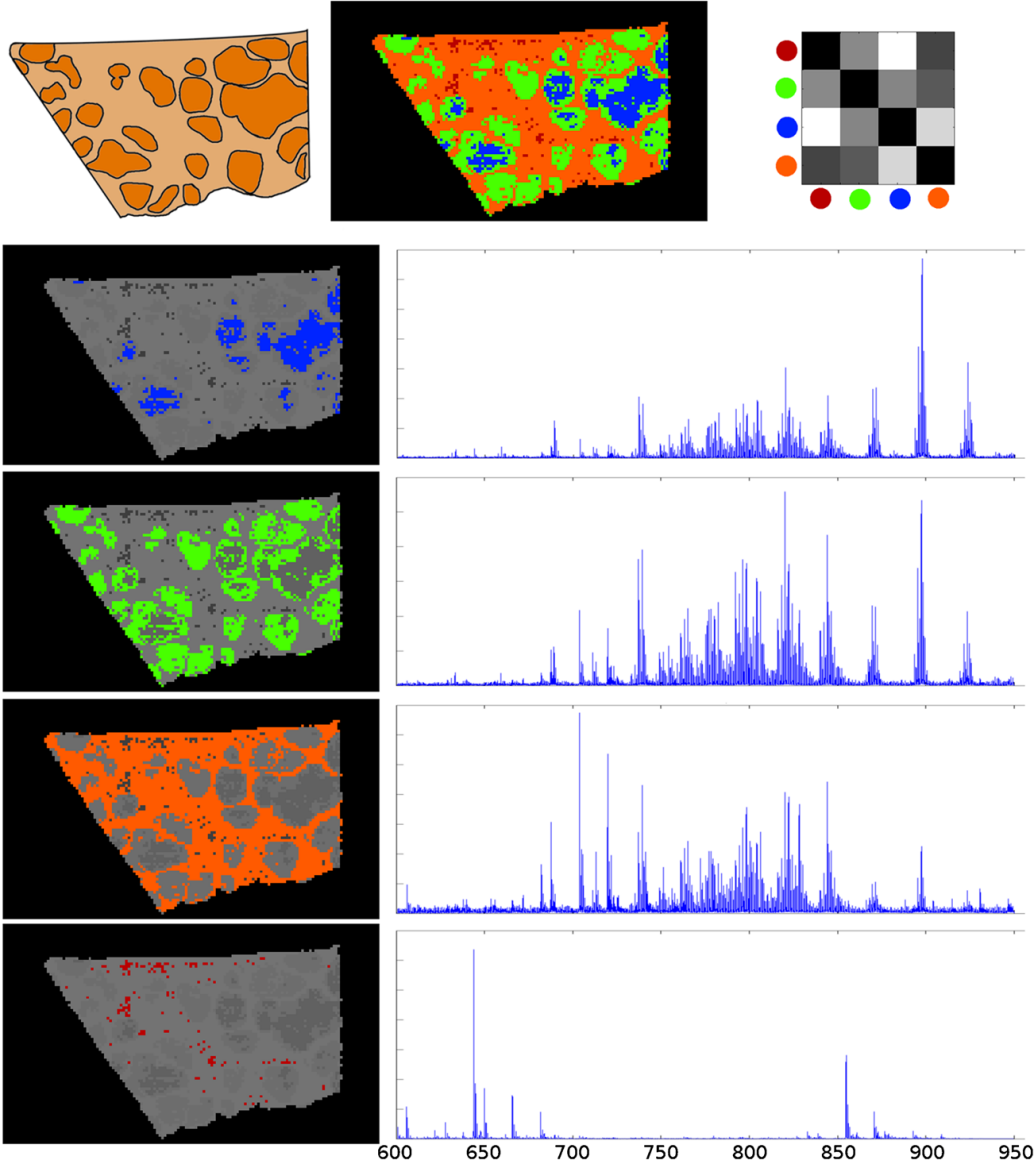
Segmentation results using random projection for dimensionality reduction followed by k-means clustering. Top row (left to right): schematic of the image; the segmentation map with each cluster shown in a unique color; the cluster centroid distances illustrating relative cluster similarity (black - identical, white - greatest dissimilarity). Lower rows: the average spectrum from each segmented pixel region provides a molecular profile for the cluster

Random projections are useful for the segmentation of mass spectrometry images because they preserve several distance metrics (e.g., Euclidean distance) [6], which allow certain types of segmentation algorithm to be applied to the low-dimensional projected data. Following projection, the pixels were clustered using the popular *k*-means algorithm that has been shown to be useful in MSI [16, 29, 1]. The segmentation results achieved using four clusters are shown in Figure 2, following projection in the spectral domain using 150 random projections, reduced from 33725 *m/z* channels. The segmentation time for *k*-means is linear in the number of dimensions, so this directly translates to a proportional reduction in the computational cost.

#### Spatial Patterns Detected by Segmentation

The image segmentation following random projection is shown in Figure 2, and shows clear delineation of the tissue section that has been determined to be consistent with histopathology. Hepatocytes are extracted from the surrounding tissue (orange), which consists mostly of fibrotic connective tissue, with the majority of hepatocytes being assigned to the same cluster (green). Interestingly, this segmentation technique identified the subpopulation of hepatocytes (blue), which were thought to be regenerating nodules, and identified the center of these nodules as being a distinct cluster. All of these assignments are in agreement with the visualization techniques of Fonville et al. [14]. Further analysis is necessary to determine the nature of the spectral differences between the clusters.

#### Spectral Properties of ROIs Derived from Segmentation

After clustering was performed on the randomly projected data, the mean spectrum for each cluster was computed from the original data. These are shown underneath the segmentation map in Figure 2. These molecular profiles show a variety of spectral differences between the regions. There is a clear difference in the relative abundances of species present, and different ions show patterns corresponding to hepatocytes (green and blue), portal areas (red), and regions of fibrotic matrix (orange). The Euclidean distance between the centroids provides an idea of how different the clusters are to each other, and this is shown in the grid in Figure 2. As this distance is based on the projection of the spectra, it is a measure of the spectral similarity between clusters, and these results indicate that the most difference is between the regenerating hepatocyte centers and the surrounding (normal) tissue, with less difference compared with the other enlarged hepatocytes.

Interpreting the spatial maps still requires input from an appropriate expert but segmentation provides a way of presenting the results from mass spectrometry imaging in a format that can be readily understood by non-mass spectrometry experts.

### Choosing the Number of Projections

We now consider how many projections are necessary to ensure that the original data is accurately represented. The search for formal upper bounds on the number of random projections is still an active field [6, 19, 12] and so we treat this as an experimental variable.

**Figure 3:**
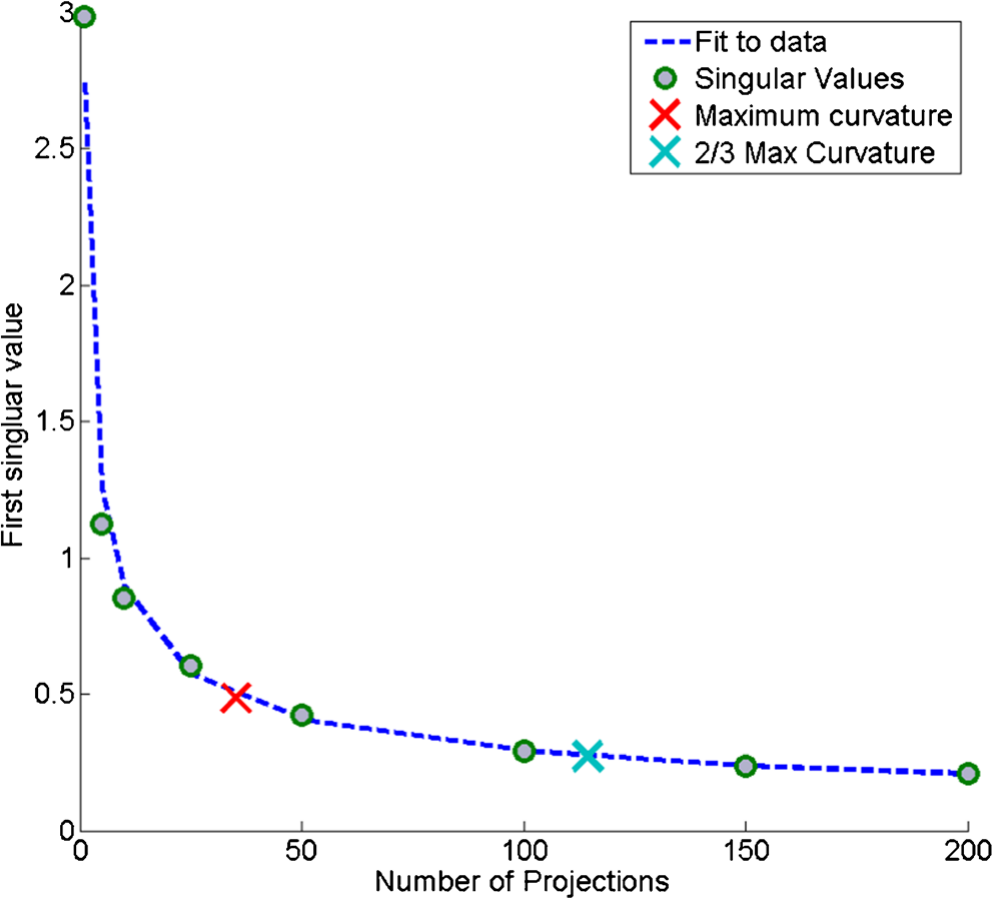
The normalized first singular value decays smoothly with increasing number of projections as less additional variance is recovered from the data. By automatically fitting a curve the point of maximum curvature can be determined

To obtain an automatic measure of how many projections to use, we inspect the change in the singular value decomposition of the projected data. The singular value decomposition (SVD) is a frequently used mathematical tool that produces a set of unique combinations of measurement variables, which can be useful for identifying patterns within data. The first singular value points along the direction of greatest data variance and, so, as more random projections are used and more of the data variance is captured, we would expect this value to stabilize, and since the random projection process dramatically reduces the dimensionality of the data, the SVD can be performed with little computational effort (there is no need to calculate the SVD of the raw dataset). Figure 3 shows the first singular value plotted against the number of random projections. The magnitude of the singular value has been normalized to the number of projections so what is seen is the variance captured per projection, which can be seen to decay as the number of projections increases. This trend was measured to be exponential and so an equation describing this curve can be automatically fitted to this trend. To obtain an automatic estimate of the number of random projections needed, first the ‘elbow’ of the curve was determined as the point of maximum curvature. This was calculated analytically from the equation describing the decay. It was then estimated that once the curvature had decreased to two-thirds of the maximum, the ‘elbow’ had been passed. The point of maximum curvature is marked with a red cross on Figure 3, at approximately 30 projections, and the estimated number of projections with a green plus at approximately 120.

An important feature of this approach is that the number of projected values can be estimated as soon as a good fit to the first singular value curve can be made, which can be made before the elbow has been reached. This can be done efficiently by taking an initial set of projections from which subsets can be drawn to generate the curve. If the total number of projections is insufficient and the elbow in the curve is not reached, further projections can be added until the elbow is seen.

### Effect of the Number of Projections on the Segmentation

**Figure 4:**
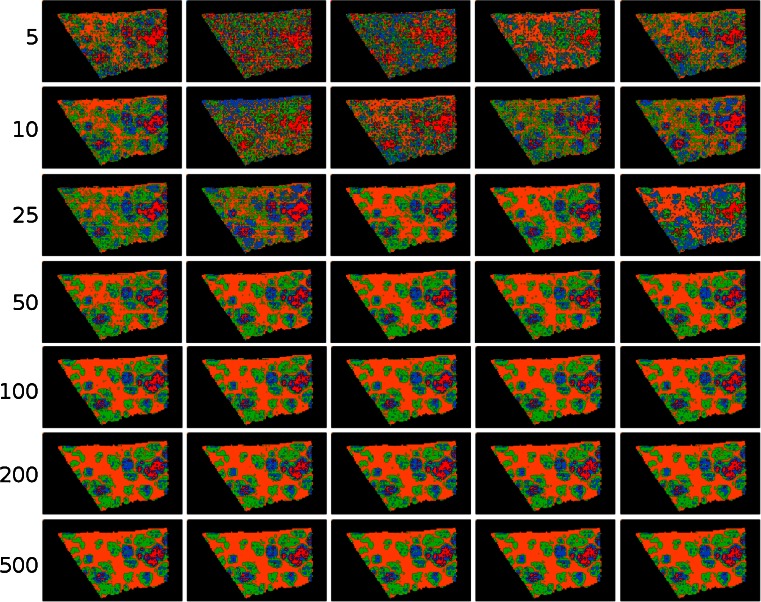
Top-to-bottom: increasing the number of projections up to around 100 increases the segmentation reproducibility; after this point, the segmentation result completely stabilizes and the same tissue patterns are produced. Left-to-right: each column is the result of a different set of random vectors. At low numbers of projections, the exact choice of projection vectors affects the results of the segmentation, whereas for higher numbers of projections, the segmentation is stable and reproducible against a different choice of projection vectors

We varied the number of projections between 5 and 200 and performed *k*-means segmentation performed for each case. The segmentation results are shown in Figure 4. As the projections statistically sample the data, we ask two related questions: how many projections are required to capture the chemical differences within the data, and how reliably can this be achieved?

We first observe that clustering on a very small number of projections *can* produce a segmentation that has some resemblance to the known tissue histology (row 1 in Figure 4) but is typically very noisy and poorly connected, and an insufficient number of random projections yields rather unstable and unreproducible clustering results.

However, experiments using a low number of projections serve to illustrate the idea that each projection samples across the whole spectrum and, therefore, a few projections capture a statistical selection of the chemical information. A small number of projections is, therefore, sufficient to identify broad trends in the data, but not the important fine details.

As the number of projections is increased, the segmentation map rapidly stabilizes. The pairwise correlation between maps produced with an equal number of random projections was calculated (see Supplementary Figure S3) and was less than 0.2 for five projections but approximately 0.9 when 200 were used. Using more than ≈100 projections yields little additional benefit, which agrees with the singular value decay shown in Figure 3 and with other results in the literature on random projections: a stable solution is reached after sufficient projections are included and the results do not significantly improve when additional projections are included [6, 19, 12]. This makes random projection a very robust dimensionality reduction technique as it is not too sensitive to the number of projections. For MALDI-MSI data, we have found that 100 to 200 are sufficient on all datasets that we have considered, which is in line with other recommendations for the number of variables to consider with classification algorithms [1]. It is useful to note the computational cost of increasing the number of projections is low as the majority of computational time is spent loading the data from disk as opposed to performing the calculations.

For a comparison with the performance of a more conventional dimensionality reduction technique, we also performed principal component analysis (PCA), which is frequently used in MSI for this purpose [3], and subsequently performed segmentation, as shown in Supplementary Figure S4. Visually, the segmentation results obtained are near-identical in both cases (with 100 RPs) with the same tissue regions identified. We also computed the correlation between segmentations following random projection and PCA, and found *P* >0.9 from 100 projections, rising slowly thereafter. This illustrates that the information required for segmentation (in particular, Euclidean distance) is preserved to the same degree by both techniques, but random projection is much more computationally efficient (Supplementary Table S1).

## Conclusions

Random projection has been shown to be a fast, repeatable, and effective dimensionality reduction tool for MSI data that can be used to enable fast and accurate segmentation. We have shown that segmentation following random projection produces results that are consistent with the known histology. As random projection permits segmentation on data that has not undergone any processing, it potentially offers a useful baseline against which the effects of further data processing can be compared. In this work, random projections were applied directly to the data without any other processing but could equally well be applied after de-noising and feature selection. Further investigation would be required into the effect this has on subsequent segmentation.

We have demonstrated the use of random projections to allow rapid segmentation using *k*-means clustering but, in principle, any segmentation or visualization method that uses the Euclidean distance metric could benefit [14, 16]. The main disadvantage of this method is that the projection matrix is, in general, not invertible. The projections are, therefore, “one-way” and the results cannot be directly interpreted in terms of the original *m/z* values. In cases where recovery of the original data is required from the projections, an orthogonalized random basis approach has previously been developed [24], which yields similar benefits for segmentation but requires additional computation.

This work has demonstrated the potential of simple random projections on MSI datasets but other spectroscopic techniques could also benefit. Related work has shown the application of random projections to Raman microscopy [23] and hyperspectral optical imaging [24], and it is therefore reasonable to expect that the results found here can be generalized to other spectral techniques. We expect there will be particular benefits in high mass-resolution mass spectrometry methods and new developments such as Rapid Evaporative Ionization Mass Spectrometry [5] or miniaturized portable spectrometers [22] that produce high-throughput data requiring real-time analysis in environments where significant computing power is not available and data transfer bandwidth may be limited. It is memory-efficient as each spectrum is processed sequentially, and is computationally inexpensive as the basis simply requires the generation of *k* random vectors. The use of computationally efficient algorithms such as random projection may be a powerful tool for the rapid classification of samples or for determining which samples require further investigation.

## Electronic supplementary material

Below is the link to the electronic supplementary material.

ESM 1 (PDF 1138 kb)
