# Supplementary material for: The Use of Random Projections for the Analysis of Mass Spectrometry Imaging Data
Source: J Am Soc Mass Spectrom. 2014 Dec 19;26(2):315–22. doi: 10.1007/s13361-014-1024-7 (PMC4320302; doi:10.1007/s13361-014-1024-7)
Supplement: Supplementary file 1 — (PDF 1138 kb) [file 13361_2014_1024_MOESM1_ESM.pdf]

The Use of Random Projections for the Analysis of MALDI Mass Spectrometry Imaging

Andrew D. Palmer, Iain B. Styles, Josephine Bunch

August 28, 2014

1 MALDI Imaging Normal Liver

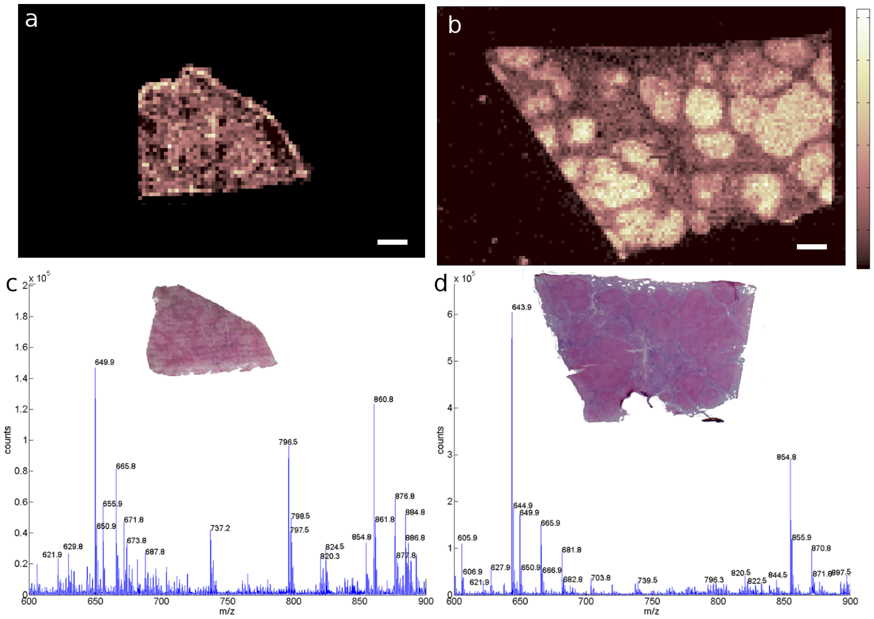

Figure 1: normal and NASH side by side showing differences in morphology

Figure 1 shows a MALDI image from a normal and a NASH diseased tissue section. The normal liver shows a much more regular morphology at the resolution of this image and there are no signs of the enlarged hepatocytes visible within the NASH tissue. The mean spectra have observable differences but it is not the aim of this work to determine specific molecules that distinguish between normal and NASH tissue, far greater sample numbers would be required than are available here.

2 Random Projection of Mouse Brain Image

The mouse brain image made publicly available by Race et al[1] underwent a workflow of random projection, using 150 projections, and k-means clustering, with five clusters. The segmentation map shown in Figure 2 delineates tissue features (white and grey matter) and the MALDI matrix background. There are two clusters that form at the boundary. This boundary phenomenon often occurs when using CHCA[3, 4]. The two boundary layers show progressively fewer peaks in the 'lipid region' m/z 700 - 900 and greater numbers of matrix clusters that are normally suppressed on tissue.

2.1 Clustering Correlation

Segmentation using k-means clustering (5 clusters) was performed on data projected with 5-200 random projections, with 5 repeat experiments being performed for each set of random projections (new projection vectors were produced each time). The resulting segmentation maps are shown in Figure 4. For each set of projections the spearman ranks correlation between the segmentation labellings was calculated and the average value for each size set of random projections is shown in Figure 3

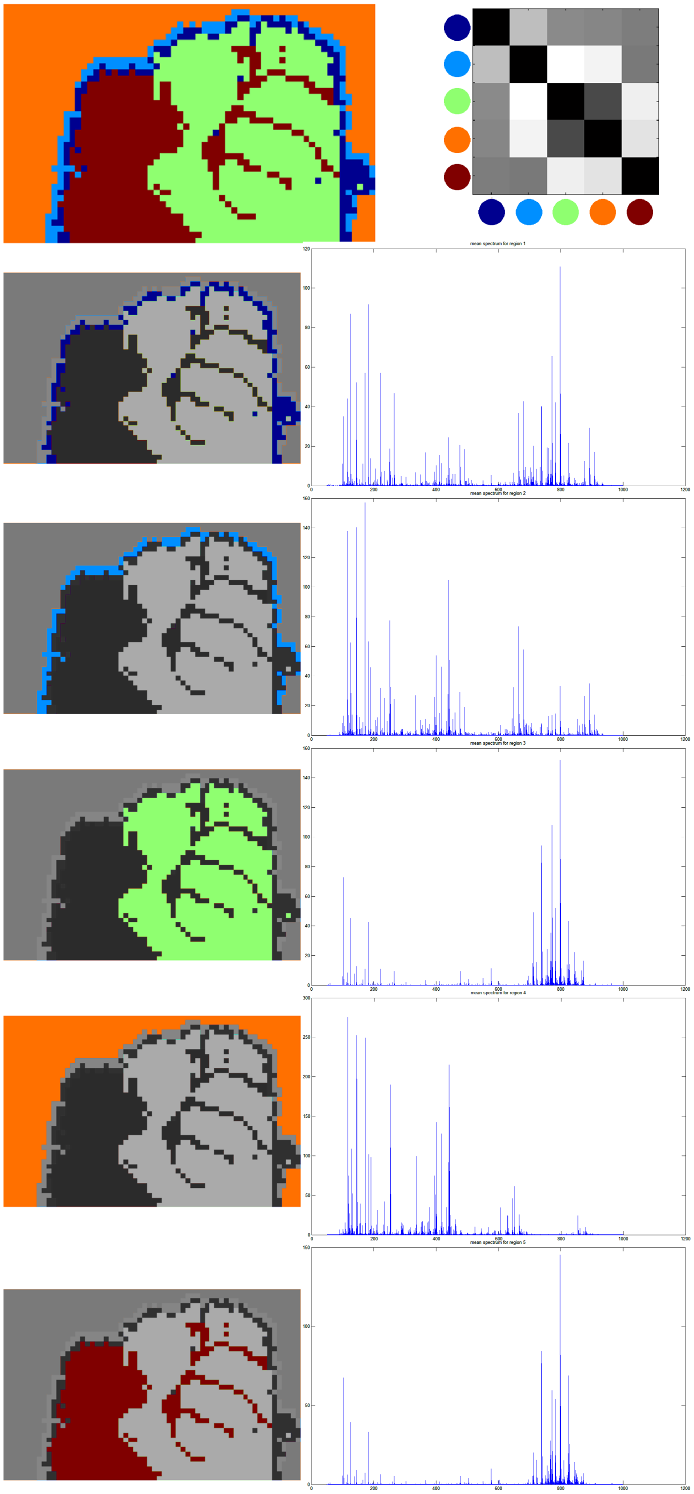

Figure 2: A workflow of random projection (150 projections) followed by k-means clustering (5 clusters) produced a segmentation that delineated the tissue section from the matrix background and separated the white and grey matter.

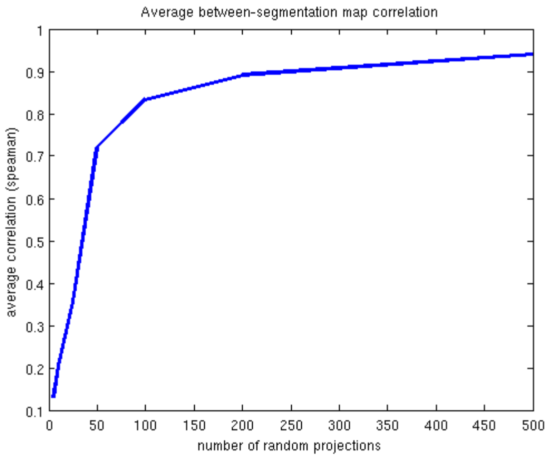

Figure 3: The pairwise correlation between segmentation results (average of 5 sets of projections) for different size random projection sets. Increasing the number of projections improves the consistency of results to over 0.9.

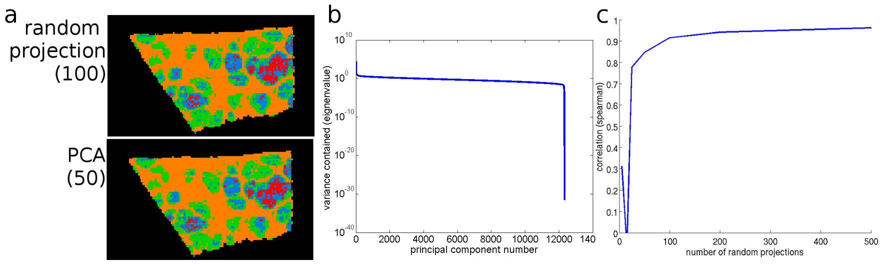

Figure 4: k-means clustering results following RP and PCA. a: segmentation maps from RP and PCA showing the identical regions are identified. b: PCA eigenvalues (variance contained) showing that only the first 50 components are non trivial. c, the average correlation (spearman, median of 5) between segmentation maps produced from dimensionality reduction using PCA or random projection

3 PCA

3.1 Equivalence of segmentation

Principal Component Analysis (PCA) was performed using the MATLAB (MathWorks, USA) function princomp. This technique produces, from within the data, a set of vectors which form an orthogonal basis for the data. The basis vectors (PCA coefficients) are ordered by the variance they contain and so only those with significant variance need to be preserved[2]. Projecting the data onto these coefficients achieves the dimensionality reduction. A near-identical segmentation is achieved from k-means applied to either randomly projected data or PCA scores (Figure 4). Figure 4.c show the average correlation between maps produced using PCA for dimensionality reduction and maps produced using varying numbers of random projections. This rapidly approaches one indicating that the tissue compartments found are identical between the two dimensionality reduction schemes. This illustrates that the information required for segmentation (in particular, Euclidean distance) is preserved by both techniques.

3.2 Computing Memory requirements for pca

Data Item (dimension)	Size (MB)
data matrix (N M) 2239.6	
mean centered data (N M)	2239.6
U (N M)	2239.6
S (M M)	4327.8
V (M M)	4327.8
Total	15,374

Table 1: Maximum Memory estimates for PCA using MATLAB princomp assuming variables are stored as 8 byte doubles.

PCA is a computationally intensive process that requires the whole dataset to be stored in memory. The requirements are summarised Stage 3 in Table 1 from Race et al[5] and re-calculated for this dataset in Table 1. This shows the maximum memory footprint of the MATLAB princomp algorithm. This was used to calculate the memory requirements for performing PCA on the MALDI-MSI from diseased human liver. Calculating the random projects took a few seconds once the data was stored in memory whilst PCA took approximately half an hour.

References

[1] Alan M. Race, Rory T. Steven, Andrew D. Palmer, Iain B. Styles, and Josephine Bunch. Memory efficient principal component analysis of large mass spectrometry imaging datasets. *OurCon Poster Presentation*, 1(1), 2012.

[2] S. Deininger, M. Ebert, A. Fütterer, M. Gerhard, and C. Röcken. Maldi imaging combined with hierarchical clustering as a new tool for the interpretation of complex human cancers. *Journal of proteome research*, 7(12):5230–5236, 2008.

[3] C.L. Carter, C.W. McLeod, and J. Bunch. Imaging of phospholipids in formalin fixed rat brain sections by matrix assisted laser desorption/ionization mass spectrometry. *Journal of the American Society for Mass Spectrometry*, pages 1–8, 2011.

[4] Rory T Steven, Alan M Race, and Josephine Bunch. para-nitroaniline is a promising matrix for maldi-ms imaging on intermediate pressure ms systems. *Journal of The American Society for Mass Spectrometry*, pages 1–4, 2013.

[5] AM Race, RT Steven, AD Palmer, IB Styles, and J Bunch. Memory efficient principal component analysis for the dimensionality reduction of large mass spectrometry imaging datasets. *Analytical chemistry*, 2013.
